# Life-stage specific transcriptomes of a migratory endoparasitic plant nematode, *Radopholus similis* elucidate a different parasitic and life strategy of plant parasitic nematodes

**DOI:** 10.1038/s41598-019-42724-7

**Published:** 2019-04-18

**Authors:** Xin Huang, Chun-Ling Xu, Si-Hua Yang, Jun-Yi Li, Hong-Le Wang, Zi-Xu Zhang, Chun Chen, Hui Xie

**Affiliations:** Laboratory of Plant Nematology and Research Center of Nematodes of Plant Quarantine, Department of Plant Pathology Pathology/Guangdong Province Key Laboratory of Microbial Signals and Disease Control, Integrative Microbiology Research Centre, College of Agriculture, South China Agricultural University, Guangzhou, People’s Republic of China

**Keywords:** Gene expression, Gene expression, Parasitology, Parasitology

## Abstract

*Radopholus similis* is an important migratory endoparasitic nematode, severely harms banana, citrus and many other commercial crops. Little is known about the molecular mechanism of infection and pathogenesis of *R. similis*. In this study, 64761 unigenes were generated from eggs, juveniles, females and males of *R. similis*. 11443 unigenes showed significant expression difference among these four life stages. Genes involved in host parasitism, anti-host defense and other biological processes were predicted. There were 86 and 102 putative genes coding for cell wall degrading enzymes and antioxidase respectively. The amount and type of putative parasitic-related genes reported in sedentary endoparasitic plant nematodes are variable from those of migratory parasitic nematodes on plant aerial portion. There were no sequences annotated to effectors in *R. similis*, involved in feeding site formation of sedentary endoparasites nematodes. This transcriptome data provides a new insight into the parasitic and pathogenic molecular mechanisms of the migratory endoparasitic nematodes. It also provides a broad idea for further research on *R. similis*.

## Introduction

The burrowing nematode, *Radopholus similis* [(Cobb, 1893) Thorne, 1949] is an important migratory endoparasitic plant nematode that was first discovered by Cobb in 1891, on the banana roots from Fiji. Previously, it was reported that *R. similis* could reproduce on more than 250 plant species including banana, citrus, black pepper, anthurium and many other commercial and ornamental plants^1^. There are two morphologically indistinguishable races of *R. similis*. The banana race is reported to parasitize banana but not citrus, whereas the citrus race infects citrus and banana^2^. *R. similis* invades and feeds in cells of the cortex of the roots. When the cells are destroyed, cavities coalesce to form red-brown lesions. As infection continues, the root tissues are converted into black mass, and the plant becomes weak, yellow and withered. The disease of banana caused by *R. similis* is known by the common name ‘toppling disease’, which causes serious losses to global banana production. Therefore, *R. similis* is classified as one of the top 10 plant parasitic nematodes (PPNs) worldwide^3^.

Females and juveniles of *R. similis* can infect but males with their weak stylets do not feed. *R. similis* can parasitize host plant successfully with the help of a large number of effectors secreted from stylet and body surface, such as cell wall degrading enzymes, antioxidant enzymes, and a variety of proteases^4–7^. Therefore, it is very important to study these effectors for *R. similis* control.

Transcriptome analysis of PPNs can help us to know more about the biochemical and molecular processes of nematode development, reproduction and interaction with plant host, and to excavate pathogenic genes effectively. Recently, transcriptome data have been obtained from many PPNs^8–17^. Transcriptome sequencing had also been performed in *R. similis* using SMART cDNA synthesis method^18^. In this study, different expressed transcripts among eggs, juveniles, females and males of *R. similis* were dig out using Illumina HiSeq 2000 sequencing. The result will lay the foundation for studying the infection mechanism and novel *R. similis* control strategies.

## Materials and Methods

### Sample preparation and RNA extraction

*R. similis* used in this study was collected from citrus roots and maintained on carrot disks at 25 °C in a dark incubator^19^. Nematodes were continuously cultured on carrot disks for more than four years before this experiment. After 50 days of culturing, nematodes on carrot disks were isolated according to Zhang *et al*.^5^. Mixed-stages nematodes were suspended in sterile water. Then, 10000 females, males, juveniles and eggs were picked up separately under a microscope. RNA of each life-stage nematodes was extracted using the RNeasy Micro Kit (Qiagen). The integrity and purity of RNAs were examined using 1% agarose gel electrophoresis and Agilent2100.

### Library construction, sequencing and data assembly, and functional classification

cDNA libraries of four life stages of *R. similis* were constructed as described previously^17^. These four libraries were sequenced by Illumina HiSeq™ 2000 (TruSeq SBS KIT-HS V3, Illumina). Data assembly and annotations were performed as described previously^17^. The full details of cDNA library construction, sequencing, data assembly used in this research were present in the Supporting Methods section in File S1. Blast2GO (v2.5.0) and WEGO (v2.5.0) were used in gene function annotation and GO term classification. Transcripts were further annotated based on the similarities with NR (release-20130408), Swiss-Prot (release-2013_03), KEGG (Release 63.0) and COG (release-20090331) databases. Pathfinder was used to get pathway annotation for each transcript.

### Sequence similarity analysis

To build a blast local database, protein sequences of *Caenorhabditis elegans*, *Ascaris suum*, *Brugia pahangi*, *Loa loa*, *M. incognita*, *M. floridensis*, *M. hapla*, *B. xylophilus* and *G. pallida* were downloaded from Wormbase (http://www.wormbase.org), *Wolbachia* (SRR944623) transcriptome data were downloaded from NCBI Genbank (http://www.ncbi.nlm.nih.gov). The transcriptome data of *R. similis* were compared with these databases using blastx or tblastn 2.3.0 (cut off E values < 10^−5^).

### Differentially expressed genes in *R. similis*

Differentially expressed genes were defined as genes which has more than 5-fold change of fragments per kilobase of transcript per million fragments mapped (FPKM) value and false discovery rate (FDR) < 10^−5^ between any two samples of eggs, juveniles, females and males. SOAP (Release 2.21) and GO-TermFinder (v0.86) were used in identification and classification of these differentially expressed genes. Then, STEM^20^ and OmicShare (www.omicshare.com/tools) were used in clustering and heatmap drawing.

### Prediction of functions genes

#### Prediction of genes coding for cell wall modifying proteins

The CAZymes Analysis Toolkit (http://mothra.ornl.gov/cgi-bin/cat/cat.cgi) was used to identify putative carbohydrate active enzymes (CAZymes) in *R. similis* based on CAZymes database. Then blastx (cut off E values < 10^−5^) was used in the prediction of genes coding for expansin-like proteins based on nematodes expansin-like protein database. Putative genes coding for cell wall modified proteins were manually collated by using NCBI blastx against NR and Conserved Domain database.

#### Prediction of other functional genes

Local database was constructed from reported antioxidant, parasitic, RNAi and chemotaxis protein sequences in *C. elegants* and PPNs downloaded from NCBI (http://www.ncbi.nlm.nih.gov). *R. similis* sequences were searched against the database using blastx (e-value cut off of 10^−5^, identity > 30%). The gene with the highest score was considered to be the most similar gene to the unigene. These aligned sequences were manually verified by using NCBI online blastx (http://blast.ncbi.nlm.nih.gov/Blast.cgi) against NR database. In order to clarify the otherness of the type and the number of predicted antioxidant genes between different parasitic types of PPNs, *A. besseyi* (http://www.ncbi.nlm.nih.gov, NCBI BioSample accession No. SAMN02420038) and *B. xylophilus* (http://www.wormbase.org, BioProject No. PRJEA64437) transcriptome data were downloaded and blasted (cut off E values < 10^−5^, identity > 30%) against the antioxidant enzyme gene database. These blast results were manually verified by using NCBI blastx (http://blast.ncbi.nlm.nih.gov/Blast.cgi) against NR database. Then these putative antioxidant genes were compared with the *R. similis* transcriptome data.

### Validation of differentially-expressed genes by qPCR

Nineteen differentially expressed genes were selected for qPCR to evaluate the accuracy of gene expression data obtained by Illumina sequencing. Gene-specific primers (Table S1) were designed using Primer 5.0. cDNA for each life stage was reverse transcribed using HiScript II Q RT SuperMix (Vazyme, China). qPCR was performed using a CFX96 qPCR instrument (Bio-Rad, Hercules, CA, USA) with AceQ qPCR SYBR Green Master Mix (Vazyme). β-actin^21^ was set as a reference gene. Relative transcript abundance was calculated using the 2^−ΔΔCt^ method^22^. All expression experiments were performed in triplicate with three biological replicates. Statistical analysis was performed using SPSS 23.0. All data were subjected to analysis by one-way ANOVA and tested for differences between treatments at a 5% level using Duncan’s Multiple Range Test (DMRT).

### Accession Number

All data generated or analysed during this study are included in this published article, its supplementary information files, and publicly available repositories. Raw RNA sequence reads were deposited at NCBI Sequence Read Archive (SRA) under accession number SRR6425985-SRR6425988.

## Results

### RNA sequencing and transcripts annotation

In this study, RNAs of eggs, juveniles, females and males of *R. similis* were sequenced using Illumina Hiseq 2000 which yields 62,440,260, 62,440,260, 62,440,260 and 62,440,260 reads respectively. 64761 unigenes were assembled in total (accumulated length of 129158666nt) with the average length of 1994nt, 3136nt for N50, which had 22,372 distinct singletons. Juveniles had the largest number of unigenes, followed by eggs, females and males (Table S2, Fig. S1). In total, 48432 unigenes were annotated by using NR, NT, Swiss-Prot, KEGG, COG, GO database respectively (Table S3). GO annotation of transcripts between different life stages have also been compared (Figs S2–S5).

### Sequence similarity analysis

**Figure 1:**
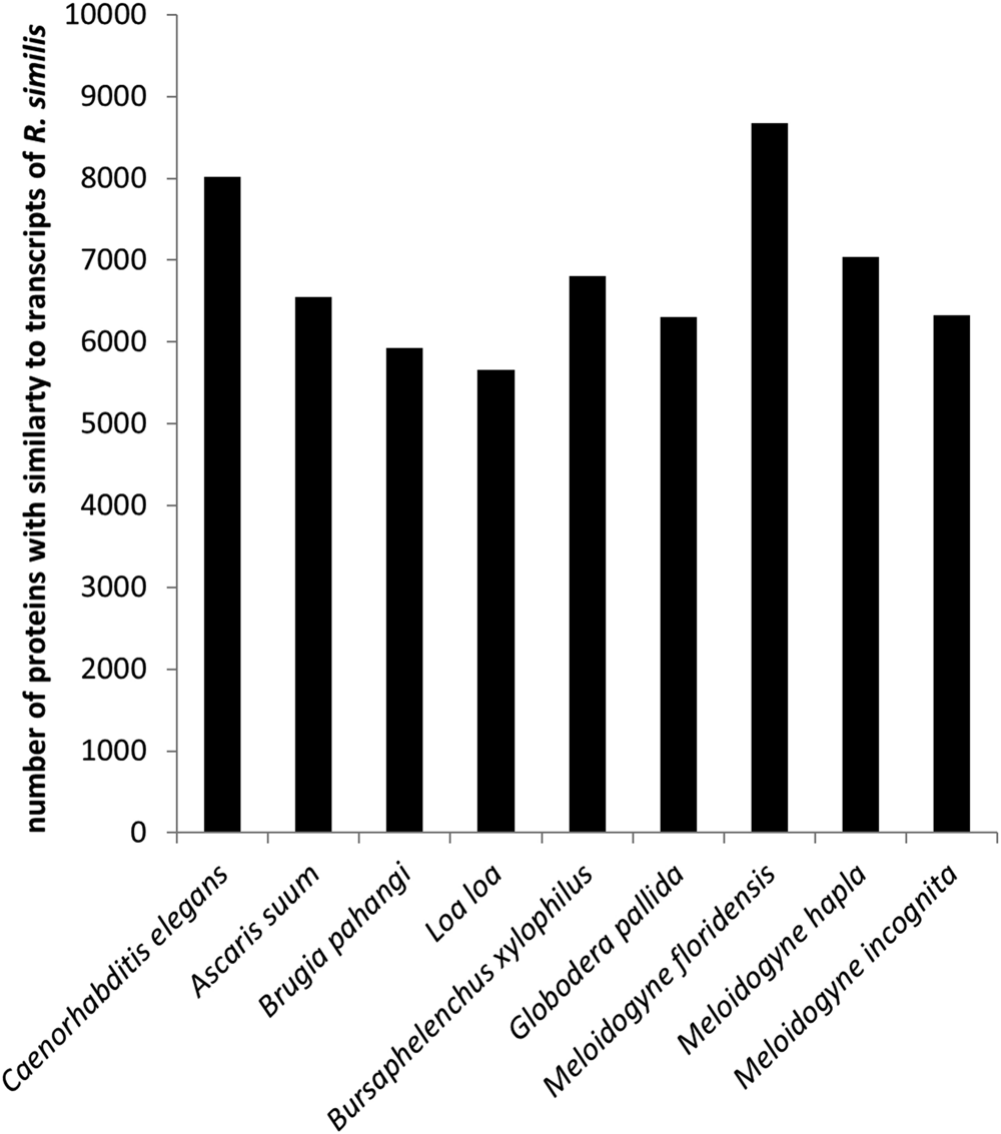
Comparison of *Radopholus similis* unigenes with proteins of *Caenorhabditis elegans, Ascaris suum, Brugiapahangi, Loa loa*, *Bursaphelenchus xylophilus*, *Globodera pallida*, *Meloidogyne floridensis*, *M. halpla* and *M. incognita*. X-axial: 9 nematodes species used in suequence alignment, Y-axial: number of proteins that have similar sequence in *R. similis*.

*R. similis* transcriptome data was blasted against the protein data of 9 nematodes including *C. elegans*, *A. suum*, *B. pahangij*, *L. loa*, *M. incognita*, *M. floridensis*, *M. hapla*, *B. xylophilus* and *G. pallida*. 8677 proteins from *M. floridensis* have similar sequences in *R. similis* transcriptome data, which were more than in other 8 nematodes (Fig. 1). 36 transcripts showing similarity with *Wolbachia* genes have been screened out. None of these sequences got the best hits with sequence in *Wolbachia* using NCBI online blastx (https://blast.ncbi.nlm.nih.gov/Blast.cgi) against NR database (Table S4). Other 744 transcripts were found to have similarity with genes from citrus (Table S5) and need further studies.

### Transcriptional changes during R. similis life cycle

**Figure 2:**
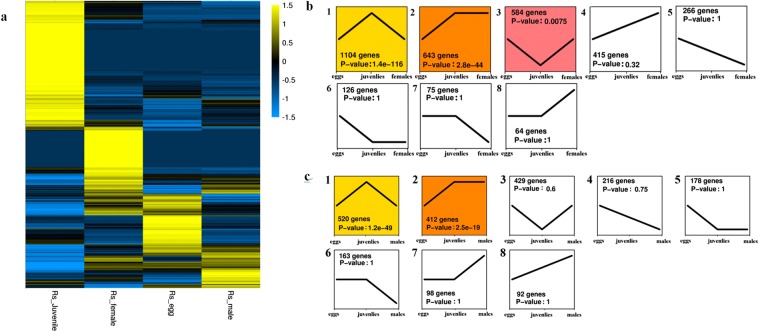
Gene expression patterns of *Radopholus similis*. (**a**) Heatmap showing all significantly differentially genes of *R. similis* (FDR < 10^−5^). Expression level for each gene was presented by normalized FPKM value. Yellow signifies an increase in expression and blue, a decrease in expression. Heatmap scale bars indicate log2-fold changes. (**b**) Transcripts in eggs, juveniles and females grouped according to temporal expression profiles. (**c**) Transcripts in eggs, juveniles and males grouped according to temporal expression profiles. Genes were divided into 16 distinct temporal profiles, using STEM^20^ software. Significant patterns (p < 0.05) are colored, profiles with the same color belong to the same cluster of profiles.

In this study, 11443 differentially expressed genes have been identified among eggs, Juveniles, females and males of *R. similis* (Fold change ≥ 5, FDR < 10^−5^) (Fig. 2a). The gene expression model profiles of *R. similis* from eggs to females and males can be clustered into eight groups (Fig. 2b,c). There were three out of eight in egg to female and two out of eight from egg to male significantly enriched expression profiles (p < 0.05) respectively.

There were 2613 genes up-regulated in juveniles compared to eggs. These genes were mainly related to immunity, digestion and infection (Fig. S6a). In addition, significantly gene up-regulations can also be found in the gluconeogenesis, insulin-like pathway and steroid hormone pathway. It has been proved that these pathways were involved in the dauer formation of *C. elegans*^23,24^. There were 3546 genes down-regulated, which were mainly related to metabolism, growth, proliferation, transcription and protein synthesis (Fig. S6b). These genes expression patterns are consistent with the process from egg to an infective juvenile stage. 1350 genes were up-regulated in females compared to juveniles, and these genes were mainly involved in protein synthesis, immunology, sex differentiation and egg laying processes (Fig. S6c). In oocyte-forming and embryonic development-related metabolic pathway can also found up-regulated genes, which can be explained as reproductive organs development and early embryonic development in females. There were 5649 differentially expressed genes during juveniles developed into males, while, 3214 genes were up-regulated in males. Most of these up-regulated genes were associated with signal transduction and energy metabolism (Fig. S6e). In contrast, 2435 down-regulated genes were significantly enriched in xenobiotics metabolism and infection pathway (Fig. S6f).

### Identifying plant parasitism genes

#### CAZYmes and cell wall degradation

Based on Carbohydrate-Active enZYmes Database, 4221 unigenes were assigned to five families of CAZYmes (File S2) using CAZymes Analysis Toolkit. 86 unigenes were found to have sequence similarity with cell wall degrading enzymes (Table S6), including fifteen sequences that could have been acquired by HGT (Table S6) and contamination from bacteria was eliminated. Among these 86 putative genes coding for cell wall modified proteins, 52 genes belong to GH5 family. 23 genes were grouped into other 6 cell wall degrading enzymes families: five genes to GH16 family, eight genes to GH30 family, two genes to GH43 and GH53 respectively, one gene to GH28 family and five genes to polysaccharide lyase. In addition, eleven genes having similarity with expansin-like protein coding genes were also found in *R. similis* transcriptome (Table 1). Among the 52 genes belonging to GH5 family, 44 genes could be divided into six clusters according to their sequence similarity.

**Table 1:** Putative genes coding for cell wall modifying enzymes in *Radopholus similis* (Rs), compared with those in *Aphelenchoides besseyi* (Ab), *A. ritzemabosi* (Ar), *Bursaphelenchus xylophilus* (Bx), *Globodera pallida* (Gp), *Meloidogyne incognita* (Mi) and *Pratylenchus coffeae* (Pc).

Family	GH5	GH45	GH16	GH30	GH43	GH53	GH28	PL3	EXPN	Total
Rs	52	0	5	8	2	2	1	5	11	86
Pc	19	0	1	1	0	2	0	2	13	38
Mi	21	0	2	6	2	0	2	30	20	81
Gp	16	0	0	0	1	2	0	7	9	35
Bx	0	11	6	0	0	0	0	15	8	40
Ab	0	4	90	0	0	0	0	0	0	94
Ar	4	7	15	0	2	0	0	0	0	28

GH, glycoside hydrolases; PL, polysaccharide lyases; EXPN, expansin-like proteins. The data of other nematodes come from previously reports^10–12,15–17^.

**Figure 3:**
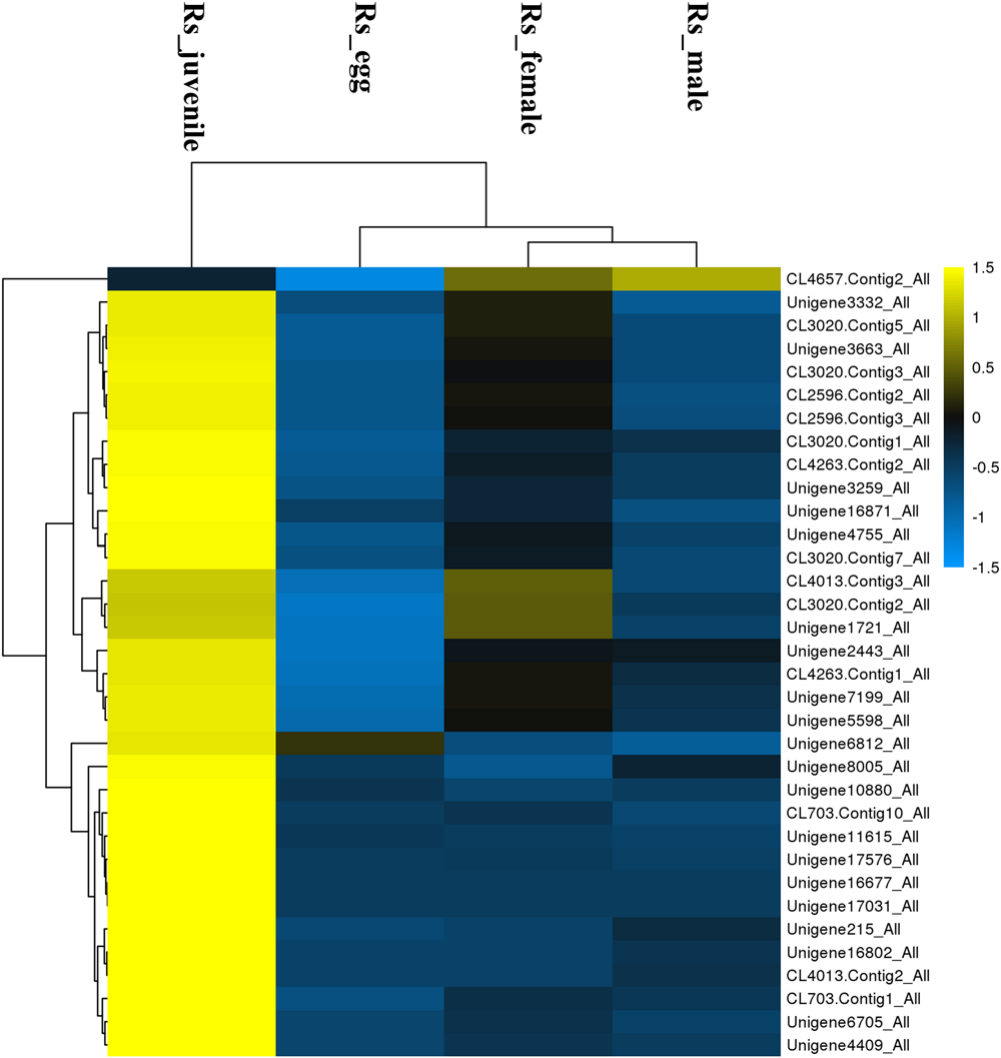
Heatmap showing the relative abundance of putative cell wall modified genes. Expression level for each gene was presented by normalized FPKM value. Yellow signifies an increase in expression and blue, a decrease in expression. Heatmap scale bars indicate log2-fold changes.

The types of cell wall modified enzymes predicted in *R. similis* were much more than in other six PPNs. There was also a difference in the type of cell wall modified enzymes between *R. similis* and Aphelenchidae nematodes, *B. xylophilus*, *A. besseyi* and *A. ritzemabosi* (Table 1). The cellulases predicted in *R. similis* belong to GH5 family, whereas most of predicted cellulases in Aphelenchidae nematodes belong to GH45 family. Only four GH5 family cellulases were predicted in *A. ritzemabosi*. However, pectin lyase was predicted only in *B. xylophilus* among these three Aphelenchidae nematodes. Expression analysis revealed that all putative genes coding for cell wall modifying protein were low expressed in eggs (Fig. 3). Most of them got the highest expression level in juveniles or females than in other stages. It’s worth noting that one predicted beta-1,4-endoglucanase (CL4657.Contig2) got the highest expression level in males. The expression pattern for this gene has been confirmed by qPCR.

### Nematode resistance to oxidative stress

102 genes were identified as antioxidant of nematodes, which can be divided into glutathione S-transferase (GST), superoxide dismutase (SOD), glutathione peroxidase and peroxiredoxin (Tables 2, S7). There were 47 sequences hit to glutathione S-transferase (GST). These predicted GSTs could be divided into eight classes: Delta, Zeta, Theta, Kappa, Sigma, Omega, Phi and Tau, most of them (n = 29) were grouped into Sigma GSTs. The expression patterns of these genes in *R. similis* were inconsistent. Each stage has several genes with the highest expression level (Fig. S7). A total of 27 genes coding for superoxide dismutase (SOD) were predicted and could be grouped into two classes: Cu-Zn SOD (n = 21) and Fe-Mn SOD (n = 6). There were eleven putative genes up-regulated in juveniles and sixteen putative genes up-regulated in adults (Fig. S7b). There were two and eight sequences hit to SODs of fungi and plant, respectively (Table S7). In the meantime, ten and eighteen sequences annotated to peroxiredoxin and glutathione peroxidase respectively. There were eight and two putative genes coding for peroxiredoxin were up-regulated in juveniles and females respectively (Fig. S7c). For genes coding for glutathione peroxidase, ten and eight were up-regulated in juveniles and adults respectively (Fig. S7d).

**Table 2:** Putative genes coding for antioxidant in *Radopholus similis* (Rs), compared with those found in *Aphelenchoides besseyi* (Ab), *Bursaphelenchus xylophilus* (Bx), *Caenorhabditis elegans* (Ce) *Globodera pallida* (Gp), *Meloidogyne incognita* (Mi) and *Pratylenchus zeae* (Pz).

Gene family	Ce	Mi	Gp	Ab	Bx	Pz	Rs
GST	44	5	13	27	55	55	47
SOD	5	3	10	5	3	20	27
peroxiredoxin	3	7	5	2	5	14	10
glutathione peroxidase	6	2	2	5	12	28	18
Total	58	17	30	39	75	117	102

Data of other nematodes taken from previously reports^10,48^. The results of the *A. besseyi*, *B. xylophilus* and *R. similis* referred to the table are shown in File S2. Glutathione S-transferase, GST; superoxide dismutase, SOD.

The species and quantity of predicted antioxidant in *R. similis* were more similar to those in *P. zeae*, than *C. elegans* and other four PPNs (*M. incognita*, *G. pallida*, *A. bessey* and *B. xylophilus*). Among all antioxidant, GST was the most abundant in these seven nematodes except for *M. incognita*. In addition, the migratory plant parasitic nematodes seemed to have more antioxidant than the sedentary plant parasitic nematodes (Table 2).

### Prediction of other parasitic related genes in *R. similis*

109 candidate genes could be classified in nine kinds of previously characterized parasitism genes of PPNs^25^ (Table 3, File S2). These parasitism genes include: calreticulin, chorismate mutase, fatty acid and retinol binding protein (FAR), 14-3-3 and SPRY domain-containing protein that affect the host signaling pathway and inhibit host defense response, ubiquitin extension protein that regulate the host ubiquitination process and proteases such as Cathepsin^25^. Among these putative parasitism genes in *R. similis*, cathepsin is the most abundant (n = 50). Besides, parasitic related genes predicted in *R. similis* were much more than in *P. coffeae*, *P. thornei* and *P. zeae*, respectively.

**Table 3:** Putative parasitic related genes in *Radopholus similis* (Rs), compared with those found in *Pratylenchus coffeae* (Pc), *P. thornei* (Pt) and *P. zeae* (Pz).

Pathogenic genes	Rs	Pc	Pt	Pz
Calreticulin	5	2	8	11
Chorismate mutase	2	1	0	0
Ubiquitin extension protein	2	0	10	5
fatty acid and retinol binding protein (FAR)	3	2	7	29
S-phase kinase-associated	8	—	—	4
Venom allergen-like protein	9	2	1	3
Cathepsin B, L, S, D	50	—	13(Cathepsin L only)	106
14-3-3	10	2	13	32
SPRY domain-containing protein	20	2	4	10

Data of other nematodes were obtained from previously reports^12,13,48^. The results of *R. similis* referred in this table were shown in File S2.

### Prediction of *R. similis* chemosensation genes

Genes coding for G proteins, signal transduction proteins and regulators in chemosensation process were predicted in *R. similis* (Table 4, File S2). Only four G proteins, GCY-35, GPA-2, GPA-3, GPA-5 and GPA-11, could found similar sequences in *R. similis* transcriptome (Table 4). Six putative signal transduction proteins have been predicted in *R. similis*, including *odr-1* and *daf-11*. *odr-1* expressed in AWC, AWB neurons which could regulate olfactory responses in *C. elegans*. *daf-11* can be expressed on both AWC, AWB and ASK neurons; it can also be expressed in ASI and ASJ and functions as a regulator of dauer formation and recovery^26,27^. Meanwhile, *R. similis* transcriptome were aligned with twelve chemosensation regulators in *C. elegans*, and only *adp-1* and *gpc-1* did not have any similar sequences in *R. similis*.

**Table 4:** Prediction of chemosensation related genes in *Radopholus similis*.

G proteins		Signal transduction		Regulators	
*gcy-35*	**Yes**	*daf-11*	**Yes**	*adp-1*	**No**
*gcy-36*	**No**	*daf-21*	**No**	*egl-4*	**Yes**
*gpa-1*	**No**	*fat-3*	**No**	*goa-1*	**Yes**
*gpa-2*	**Yes**	*ocr-2*	**Yes**	*gpc-1*	**No**
*gpa-3*	**Yes**	*odr-1*	**Yes**	*grk-2*	**Yes**
*gpa-5*	**Yes**	*odr-4*	**No**	*kin-29*	**Yes**
*gpa-6*	**No**	*odr-8*	**No**	*let-60*	**Yes**
*gpa-8*	**No**	*osm-9*	**Yes**	*npr-1*	**Yes**
*gpa-10*	**No**	*tax-2*	**Yes**	*osm-9*	**Yes**
*gpa-11*	**Yes**	*tax-4*	**Yes**	*sdf-13 (tbx-2)*	**Yes**
*gpa-13*	**No**	*unc-101*	**No**	*tax-6*	**Yes**
*gpa-14*	**No**			*ttx-4 (pkc-1)*	**Yes**
*gpa-15*	**No**				
*odr-3*	**No**				
*odr-10*	**No**				
*str-2*	**No**				

### Genes involved in RNAi pathway

To predict genes involved in the RNAi process, transcriptome data of *R. similis* was blasted against 72 proteins involved in *C.elegans* RNAi process (File S2). The result showed that putative RNAi genes were annotated to endogenous pri-miRNA formation and exportation genes *drsh-1*, Dicer complex and RISC (RNA-induced silencing complex) component genes *dcr-1*, *drh-1*, siRNA amplification genes *ego-1*, *mut-7*, RNAi intercellular spreading gene *rsd-3* and RNAi negative regulation genes *eri-1/6/7*. No sequence was found to have similarity with *sid-1/2*, which facilitate exogenously double-stranded RNA enter cells.

Maybe, there were only few genes associated with small RNA synthesis, dsRNA uptake and spreading, RNAi inhibitors in *R. similis* compared to animal parasitic nematodes including *A. suum* and *B. malayi*. However, the number of putative nuclear RNAi effectors in *R. similis* was similar to *A. suum* and *B. malayi*. Moreover, *R. similis* and other PPNs including *B. xylophilus*, *M. hapla*, *M. incognita* and *G. pallida* were similar in type of putative RNAi genes (Table S8).

### Validation of gene expression profiles by qRT-PCR

**Figure 4:**
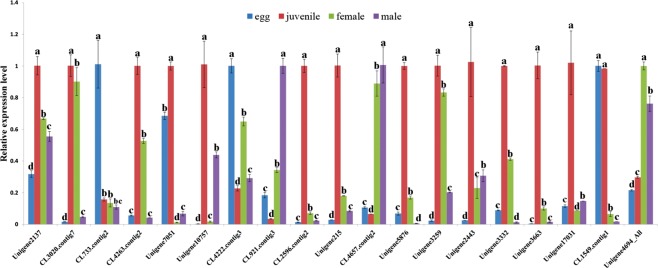
qPCR validations of 19 genes in *Radopholus similis* transcriptome data. *Actin* was used as the reference gene to detect the expression levels of 19 genes. The X axis shows the 19 genes in eggs, juveniles, females and males, the Y axis shows the relative expression of these genes in four life stages. The expression is relative to the stage which has the highest expression level. For each gene, different letters indicate significantly differences (p < 0.05) between different life stages.

To elucidate the expression profiles of this sequencing, nineteen differentially-expressed genes were chosen for qRT-PCR. The stability of the reference gene was test (Table S9). These genes were from various functional categories including cell wall degradation, antioxidant and biological progress etc. (Table 5). Among these genes, thirteen genes shown significantly higher expression level in juveniles than other life stages (p < 0.05), which were annotated as cell wall degradation genes, FAR and two function unknown protein. Two genes shown significantly higher expression levels (p < 0.05) in males than other life stages were predicted to be involved in cell wall degradation and antioxidant. Three genes were highly expressed in eggs which annotated as DNA-binding protein, calreticulin and SPRY domain containing protein. Furthermore, one putative gene coding for serine carboxypeptidase has also been confirmed to have a significantly higher expression level in females (p < 0.05) than in other life stages (Fig. 4). This independent experiment and statistical analyzed data revealed the reliability of the sequencing data.

**Table 5:** Differentially-expressed genes used in qPCR validation.

Gene_ID	Functional annotation	FPKM value for each sample			
		Eggs	Juveniles	Females	Males
CL2596.Contig2_All	xylanase	0.9202	93.2649	38.0198	4.476
CL3020.Contig7_All	GHF5, endo-1,4-beta- glucanase precursor	1.9736	106.9242	32.0546	5.9293
CL4222.Contig3_All	DNA-binding protein HEXBP	38.0629	8.8376	24.4024	24.1772
CL4263.Contig2_All	GHF5, endo-1,4-beta- glucanase precursor	3.6	603.8614	175.2951	51.2069
CL4657.Contig2_All	beta-1,4-endoglucanase	2.5918	69.228	111.9429	180.4387
CL733.Contig2_All	calreticulin	259.9156	233.3356	148.5939	166.7796
Unigene10757_All	-	0.5019	74.9161	35.9321	1.7233
Unigene2137_All	translationally controlled tumor protein	1020.11	3237.259	2323.226	1714.422
Unigene215_All	expansin	0	28.2355	1.6438	2.1128
Unigene2443_All	Xylanase	0.2885	44.6632	15.4155	9.1639
Unigene3259_All	pectate lyase	2.2263	113.7185	26.4045	12.0389
Unigene3332_All	expansin B3	2.1638	53.2293	25.7209	0.4643
Unigene3663_All	xylanase	1.6641	535.4245	204.4887	19.284
Unigene5876_All	-	2.5213	266.6904	79.9248	4.3285
Unigene7051_All	fatty acid and retinol binding protein 2	4.5902	174.7749	91.6568	126.329
CL1549.Contig1_All	SPRY domain containing protein	8.41	0.6804	3.2876	0.1761
CL921.Contig3_All	manganese superoxide dismutase	12.7638	8.1537	16.0629	16.7992
Unigene17031_All	expansin 2 precursor	0	5.3338	0	0
Unigene4694_All	serine carboxypeptidase	44.3317	48.1264	107.6178	47.8246

## Discussion

*R. similis* is an important migratory endoparasitic nematode, the morphology and biological characteristics of this nematode in different developmental stages were different. This is the first study on transcriptome of *R. similis* in eggs, juveniles, females and males separately. The number of unigenes obtained from juveniles sequencing were highest, followed by eggs, females and males. Total 64761 unigenes were obtained from *R. similis* transcriptome including 11443 differentially expressed genes. The expression patterns of differentially expressed genes were consistent with the biological functions of each life stage, and reflect the changes in the morphological and infectious ability of this nematode. The infection-related genes were up-regulated at juvenile stage and down- regulated at male stage. The genes associated with genital development such as gonads were up-regulated at female stage.

Transcriptome sequencing has been used in effectors research of PPNs^9,28,29^. These effectors include cell wall degrading enzymes, antioxidant enzymes and other proteins involved in resistance to the host defense responses. Many genes coding for cell wall modified enzymes were predicted in *R. similis*, as compared to other nematodes especially in the GH5 family. There were six clusters containing 44 sequences in the predicted GH5 family. It is speculated that there are lots of alternative splicing sites in the GH5 family genes of *R. similis*. As one kind of migratory endoparasitic nematode, *R. similis* can infect more than 250 plant species. To loosen cell walls of different plants and migrate in plant tissue, *R. similis* have evolved a variety of cell wall degrading enzymes. Only one predicted gene, coding for cell wall modified enzyme was highly expressed in males indicted that males also needed to degrade the cell wall in order to facilitate its movement in host tissue. *R. similis* was under the pressure of the reactive oxygen species produced by the host all the time. So, they must synthesize peroxidase to neutralize ROS in host plant^28^. All putative genes coding for SODs, glutathione peroxidases, peroxiredoxins and part of genes coding GSTs were highly expressed in juveniles and adults. These expression patterns were consistent with the biological characteristics of *R. similis*. It was reported that a glutathione peroxidase with signal peptide was secreted to the body surface of nematodes against the host’s ROS. This gene was expressed during the whole infective stages of *G. rostochiensis*^29^. It can be speculated that these genes predicted in *R. similis* may also be involved in the resistance to the host ROS process. Besides, some genes coding for GSTs, highly expressed in eggs of *R. similis*, may play a role in xenobiotics metabolism^30^. Six kinds of putative effector genes have also been identified in *R. similis*. These effectors have been reported previously to play a role in host signaling regulation, host defense response inhibition and degradation of the host protein process^31–34^. Many sequences are identified as genes coding for cathepsin, which play a role in degrading host cell wall proteins and host guard proteins, predigesting proteins before uptake or protein digestion in the body^25^.

By comparing the types and numbers of pathogenic genes in the PPNs of different parasitism types, it can be confirmed that the parasitic modes of the nematodes are closely related to the type and expression pattern of their pathogenic genes. For *R. similis*, the number of putative genes coding for cell wall modified enzymes, peroxidases and proteases was higher than that of the sedentary endoparasite nematodes. On the other hand, no gene identified as effector required for giant cell or syncytium formation induced by root knot and cyst nematode such as 7E12^35^ CEPs CLE peptide^36^ and 16D10 CLE^37^ were found in *R. similis*. Similarly, no sequences similar to MAP-1 involved in host recognition of root-knot nematode^38^ were found. In addition, no sequences similar of disrupt host defenses effectors as 10A06, Hs19CO7 and 30C02^39–41^ from cyst nematodes were found in *R. similis*. This nematode probably don’t have these effectors which have been reported in sedentary endoparasitic nematodes. The difference between *R. similis* and sedentary endoparasite nematodes also exist in the expression patterns of pathogenic genes. Predicted pathogenic genes in *R. similis* were highly expressed in both juveniles and females, while these genes in sedentary endoparasite nematodes mainly expressed highly in juveniles^28,29,40^. All these differences between *R. similis* and sedentary endoparasitic nematodes may be related to the different parasitic strategies. As a migratory parasitic nematode, *R. similis* move and feed in the hosts. It is always necessary to degrade host cell walls which directly lead to the necrosis of the host cell rather than altering the structure of the host cell and maintaining the cell activity as sedentary endoparasite nematodes do. *R. similis* also needs to face more oxidative stress from the host during migrating and feeding, and therefore require more peroxidases. The similar phenomenon has been reported previously in *P. zeae*^42^.

No putative genes coding for cell wall modified enzymes in GH45 family was found in *R. similis* and other nematodes parasitizing plant underground tissue. At present, GH45 family genes are only reported in Aphelenchoidea nematodes for PPNs^43^. It is supposed that the GH45 genes in the nematode may be horizontally transferred from fungus^44^. Nematodes parasitizing aboveground parts of plants can feed on fungi to complete their life cycle. Therefore, some species of these nematodes may acquire GH45 genes during long-term feeding on fungi. While, *R. similis* and other nematodes parasitizing plant underground tissue can only feed on live plant tissue, therefore these nematodes have no GH45 gene. In addition, antioxidant enzymes predicted in *R. similis* were more than in *A. besseyi* and *B. xylophilus*, which may be due to the complex soil environment for *R. similis* to survive.

*C. elegans* larvae will stop developing and form dauer larva to increase the adaptability to the environment when it is perceived the large number of populations, the insufficient food supply, or the unsuitable ambient temperature^24^. The formation and recovery of dauer larva is regulated by signal transduction pathways such as guanylyl cyclase pathway, TGFβ-like pathway, Insulin-like pathway and steroid hormone pathway. At the same time, change has also been found in the energy acquisition in the dauer larva stage of *C. elegans*. The metabolism of dauer larvae is adapted to utilize internal energy reserves, predominantly lipid in the form of triglycerides, but also glycogen^45^. Dauer larva in *R. similis* has not been reported. However, *R. similis* could survive for six to eleven months without host^46,47^. When infect host, *R. similis* complete its life cycle in 18 to 20 days at 24 to 27 °C^48^. In this study, we found that genes related to dauer larva energy metabolism in the gluconeogenesis pathway, and the genes involved in signal transduction of insulin-like pathway and steroid hormone pathway were up- regulated in the juvenile. This results indicated that *R. similis* has the basis of signal regulation and energy metabolism of develops into a dauer-like larva. It is speculated that the development of juveniles to adults stop when the external environment is not suitable for their survival and develop into dauer-like larva to overcome unsuitable environment.

By comparing the predicted RNAi related genes between *R. similis* and other nematodes, we found that some genes involved in the *C. elegans* RNAi pathway are conserved in nematodes such as *smg-2/6* for dsRNA uptake and spreading, *xpo-1/2* for endogenous pre-miRNAs formation and transportation, and *xrn-2* for RNAi inhibition^10^. In *C. elegans*, SID-1/2 is a key transmembrane protein for exogenous dsRNA and Exo-siRNA entry into cells^49,50^ and RNAi delivery between cells is required with the assistance of RSD-2/3/6^51^. However, none of these genes got similar sequence in *R. similis*. In addition, the number of predicted RISC genes in *R. similis* was less than in *C. elegans*. We did not find all genes that were important for entering and spreading of dsRNA. But RNAi can also been observed after treated with genes specific dsRNAs^7,34^, suggesting that alternative proteins or similar dsRNA propagation mechanisms or a class of effectors that play a role in both endogenous and exogenous RNAi^52^. *R. similis* may also have the similar mechanism with root-knot nematodes.

HGT from bacteria and fungi to PPNs are quite common^53–59^. In this study, 15 predicted cell wall degrading genes horizontal transfer from fungi and bacteria have been found. We also found that 744 transcripts have sequence similarity with genes from citrus. So far, only three such candidate genes from host plant been reported in *G. rostochiensis*^60^. Only some candidate genes giving that expression have been verified in this study and other genes deserve further investigation.

This study, first time revealed the gene expression of *R. similis*, excavated a variety of pathogenic genes. The study is crucially important for examining the parasitic and pathogenic molecular mechanisms of migratory endoparasitic plant nematodes (such as *R. similis*), and their interaction with hosts.

## Supplementary information

Supporting methods, supplementary tables and supplementary figures

Supplementary Dataset 1

Supplementary Dataset 1
