# Supplementary material for: Life-stage specific transcriptomes of a migratory endoparasitic plant nematode, Radopholus similis elucidate a different parasitic and life strategy of plant parasitic nematodes
Source: Sci Rep. 2019 Apr 18;9:6277. doi: 10.1038/s41598-019-42724-7 (PMC6472380; doi:10.1038/s41598-019-42724-7)
Supplement: Supplementary file 1 — Supporting methods, supplementary tables and supplementary figures [file 41598_2019_42724_MOESM1_ESM.pdf]

Life-stage specific transcriptomes of a migratory endoparasitic plant nematode, *Radopholus similis* elucidate a different parasitic and life strategy of plant parasitic nematodes

Xin Huang, Chun-Ling Xu, Si-Hua Yang, Jun-Yi Li, Hong-Le Wang, Zi-Xu Zhang, Chun Chen，Hui Xie*

Laboratory of Plant Nematology and Research Center of Nematodes of Plant Quarantine, Department of Plant Pathology Pathology/Guangdong Province Key Laboratory of Microbial Signals and Disease Control, Integrative Microbiology Research Centre, College of Agriculture, South China Agricultural University, Guangzhou, People's Republic of China.

*correspondence and requests for materials should be addressed to H.X. (email: xiehui@scau.edu.cn)

Supporting methods

cDNA library construction, sequencing, data assembly

After total RNA extraction and DNase I treatment, magnetic beads with Oligo (dT) were used to isolate mRNA from total RNA. The mRNAs mixed with fragmentation buffer were broken into short fragments at room temperature. Then, cDNAs were synthesized using the mRNA fragments as templates. Short fragments of cDNAs were purified, and single nucleotide A (adenine) was added. The cDNAs were then ligated with adapters, and suitable fragments were selected as templates for PCR amplification. Agilent 2100 Bioanalyzer (Agilent Technologies, Palo Alto, CA) and StepOnePlus Real-Time PCR System (Applied Biosystems) were used for the quantification and qualification of the sample libraries. These libraries were sequenced using an Illumina HiSeq™ 2000. In the bioinformatics analyses, adaptors and low-quality reads were removed. Then, transcripts were assembled with short reads using Trinity (release-20130225).

Table S1 Primers used in qPCR validation

gene ID	primers	
CL3020.Contig7	qCL3020-7f	GGCATGTCCCTGTTCTGGAG
	qCL3020-7r	GGGACGGGTTGTTCAGGTAG
Unigene5876_All	q5876f	CAGTTGTCGCCCTGCATCTA
	q5876r	AGAGGACGCAATGTACCACC
Unigene3332_All	q3332f	CGGCTCCAGGTAGTTGAAGG
	q3332r	TGTGCAAGGGGATCTGTGTC
CL4657.Contig2_All	QC4657-2F2	GTTGGTCTCTGTCGGGTTCA
	QC4657-2R2	CTGGGCAGTGGGCTTCTT
Unigene2137	QU2137F	AATGAACTGCTACGAAGA
	QU2137R	CCAGATGTCCGATAACTT
CL4222.Contig3	QC4222-3F	CGTTGAGTTGAGAAGAGAGA
	QC4222-3R	ATGGCATCGGTAGCATTC
Unigene3663	QU3663F	AGACCATTCAGGGCTTTG
	QU3663R	TGGTTGGAATTGGAGTTG
CL2596.Contig2	QC2596-2F	TCAACTTCGTGAAGAACTAC
	QC2596-2R	GCTTAGCGAATGGTCAAA
Unigene3259_All	QU3259F	AGACTGGTCCTTAGCAACA
	QU3259R	AAGAGTCGCCGTAGTTCA
CL1549.Contig1_All	QC1549-1F	CTCGGCGTTTGCGTTGTG
	QC1549-1R	CCGCTGGCAGTGTCCTTC
Unigene10757_All	q10757f	AATGAATCCCCACTCGCTGTT
	q10757r	CTGTTCGCTCGCAGTTTCTT
CL733.Contig2_All	QC733-2F	AAGCAAAGAACCACCTGA
	QC733-2R	CGGCAGTAGTTCCCAAT
CL4263.Contig2_All	qCL4263-2f	CTACGTGATCGTGGACTGGC
	qCL4263-2r	GTACAGGATGTGCGGGTAGG
CL921.Contig3	QC921-3F2	CTAATGCTTCTTGTGTCTTCT
	QC921-3R2	CTTCCTGACTTGAGTTATGATT

Unigene7051_All	QU7051R	TTACTGCTCACTCAATGC
	QU7051R	TCAATCGGTCGTAGAACT
Unigene2443_All	QU2443F	GAGCATCGGAAACACAAA
	QU2443R	GTTCAACAGTTGGTCAGT
Unigene215_All	QU215F	GGTCGTGGACGCCTTCTTG
	QU215R	TGGCTGTTTTGTGCCTTTGC
Unigene17031_All	QU17031F	TCACCGCCACCAACTTCTGC
	QU17031R	TCCTTGACATTGCTTGCCATCC
Unigene4694_All	QU4694F	TGAATGTTAGAAACCCAATCAAAG
	QU4694R	CACTACGACACATTGAACCCCA
Actin	Actin-f	GAAAGAGGGCCGGAAGAG
	Actin-r	AGATCGTCCGCGACATAAAG

Table S2 Evaluation of assembly quality of four life stages of *Radopholus similis* transcriptome sequencing.

Sample	Total Number	Mean Length(nt)	N50	Distinct Clusters	Distinct Singletons
egg	51,981	1792	2999	31,388	20,593
male	39,599	1537	2497	21,710	17,889
Juvenile	55,176	1612	2738	31,544	23,632
female	45,129	1467	2563	23,109	22,020
All	64,761	1994	3136	42,389	22,372

Table S3 Functional annotation for *Radopholus similis* transcriptome against NR, NT, Swiss-Prot, KEGG, COG and GO database.

Sequence File Name	NR	NT	Swiss-Prot	KEGG	COG	GO	ALL
R.similis.fa	47,595	13,296	43,010	39,448	25,017	30,444	48,432

Table S9 Stability analysis of actin in different life stages of *Radopholus similis*.

	GJ- egg	GJ- juvenile	GJ-female	GJ-male
Average Ct-1*	27.9274	28.15754	27.6127	28.0742
Average Ct-2*	22.2646	23.0781	22.7527	22.9543
Average Ct-3*	21.0669	21.3497	21.1092	21.2655
Average Ct values of the three experiments	23.7529	24.1951	23.8249	24.0980
SD of average Ct		0.1838		
C.V (%)		0.7671		

*1, 2, 3: represents three independent experiments.

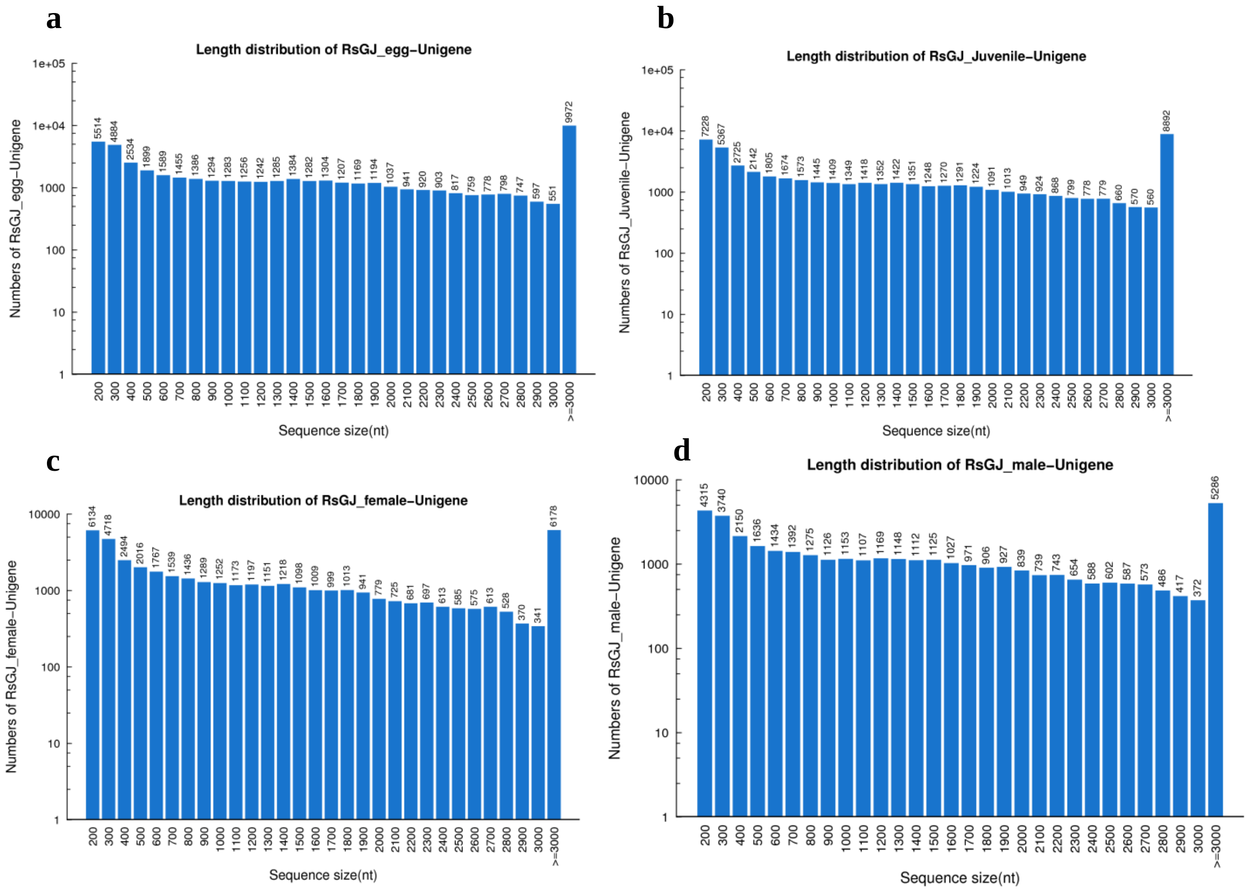

Figure S1 Length distribution of the *Radopholus similis* transcriptome

(a) Unigene length distribution of *R.* eggs. (b) Unigene length distribution of *R. similis* juveniles. (c) Unigene length distribution of *R. similis* female. (d) Unigene length distribution of *R. similis* male.

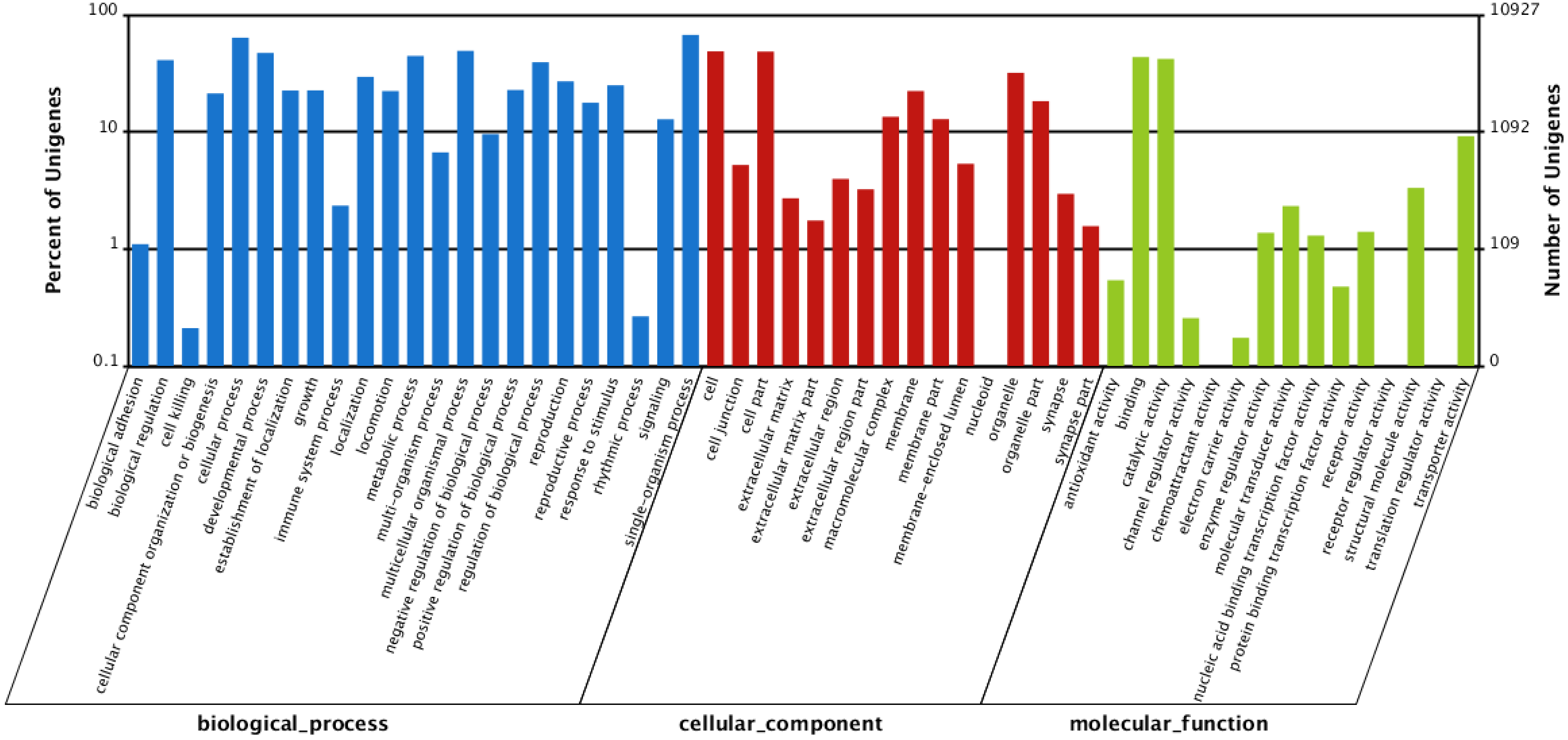

Figure S2 GO classification of differentially expressed genes between eggs and juveniles of *Radopholus similis*.
GO functions are shown on the X-axis. The right side of the Y-axis shows the number of genes with the GO function, and the left side shows the percentage.

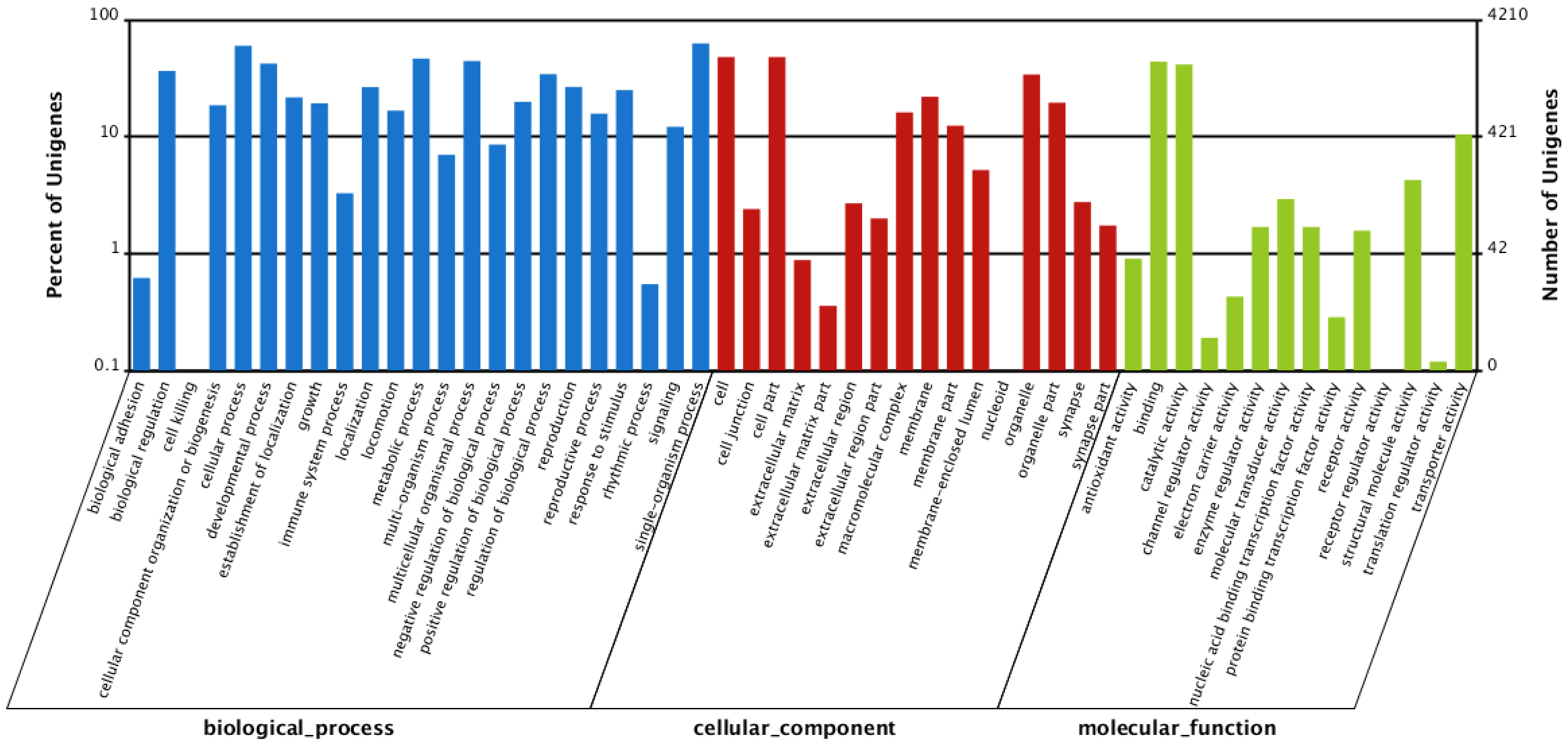

Figure S3 GO classification of differentially expressed genes between juveniles and females of *Radopholus similis*.

GO functions are shown on the X-axis. The right side of the Y-axis shows the number of genes with the GO function, and the left side shows the percentage.

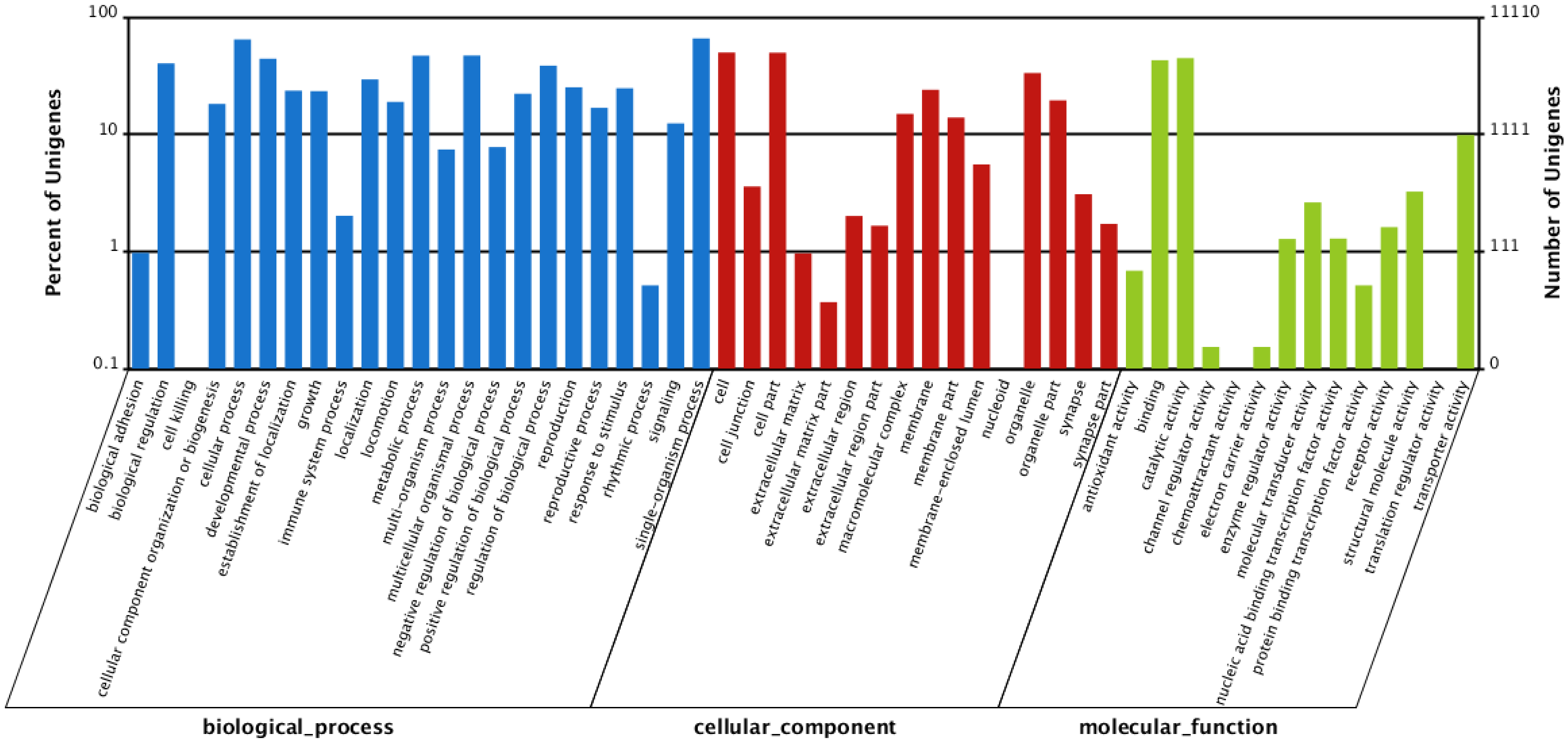

Figure S4 GO classification of differentially expressed genes between juveniles and males of *Radopholus similis*.

GO functions are shown on the X-axis. The right side of the Y-axis shows the number of genes with the GO function, and the left side shows the percentage

.

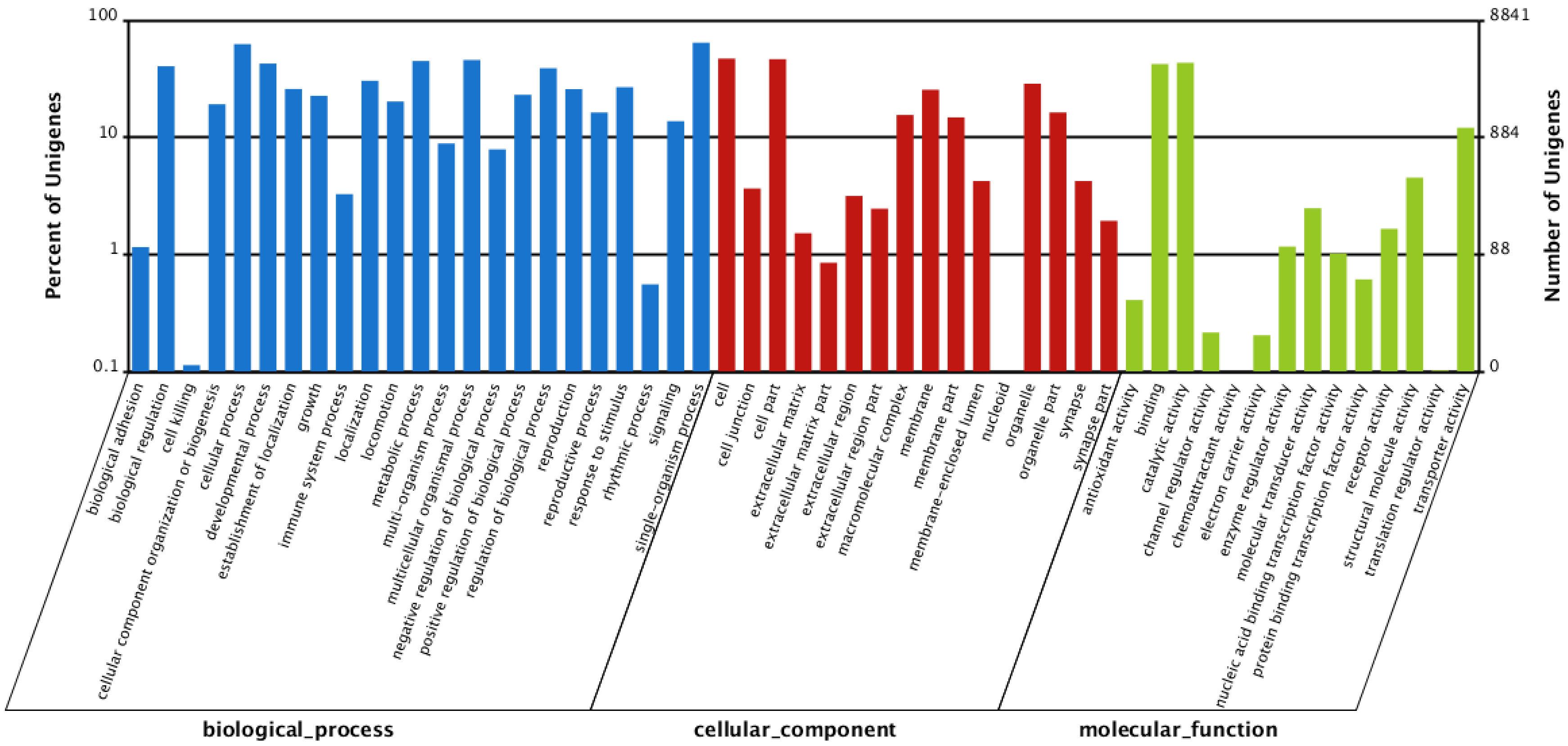

Figure S5 GO classification of differentially expressed genes between males and females of *Radopholus similis*.

GO functions are shown on the X-axis. The right side of the Y-axis shows the number of genes with the GO function, and the left side shows the percentage

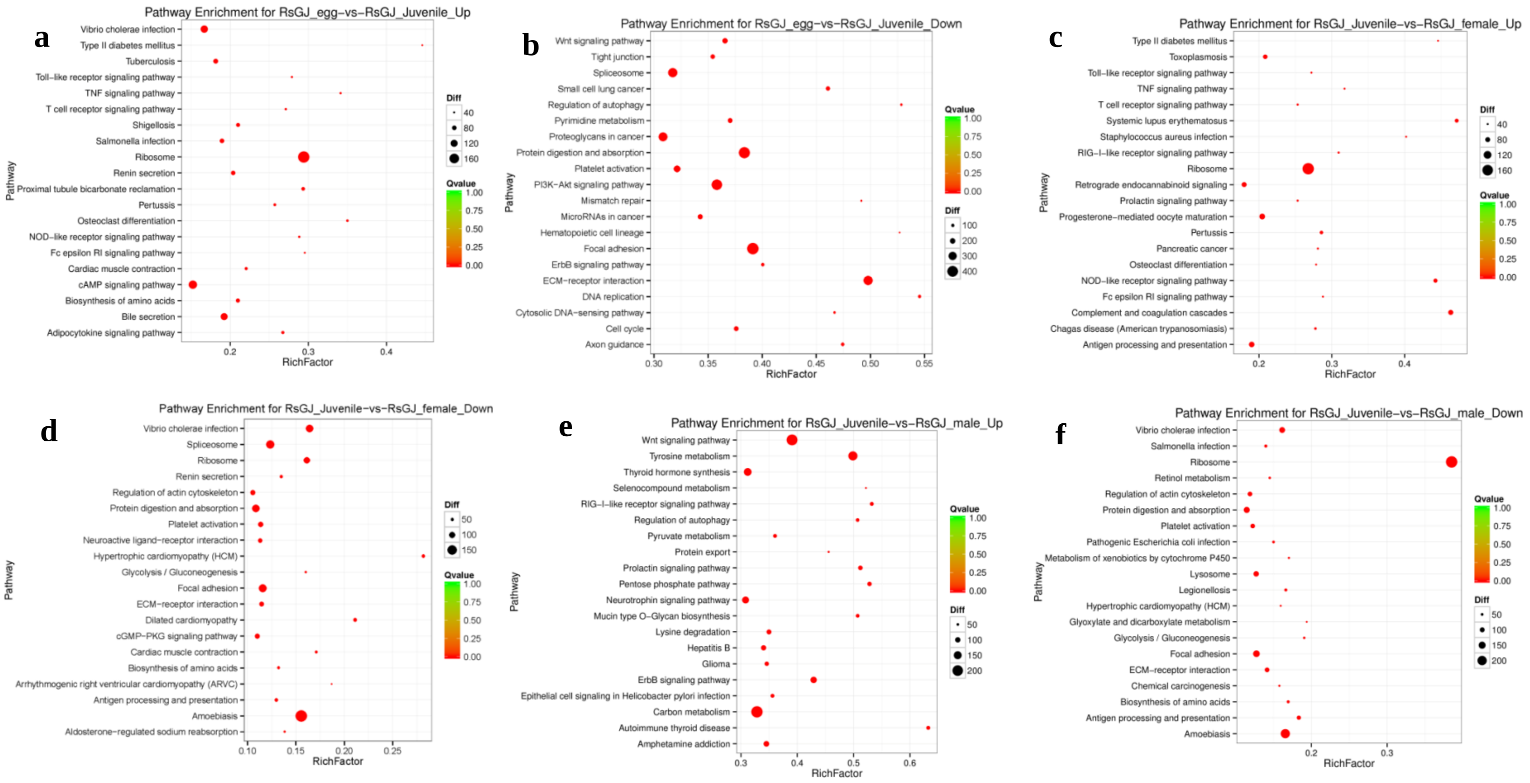

Figure S6 KEGG pathway enrichment of differentially expressed genes in *Radopholus similis*

(a) KEGG enrichment of up-regulated genes in juveniles compared with eggs. (b) KEGG enrichment of down-regulated genes in juveniles compared with eggs. (c) KEGG enrichment of up-regulated genes in females compared with juveniles. (d) KEGG enrichment of down-regulated genes in females compared with juveniles. (e) KEGG enrichment of up-regulated genes in males compared with juveniles. (F) KEGG enrichment of down-regulated genes in males compared with juveniles. X-axis: Rich factors of enriched KEGG pathway. Y-axis: Pathways annotated in the *R. similis* transcriptome. The color change from green to red signifies the change of q value from 1 to 0. Diff means the number of differentially expressed genes.

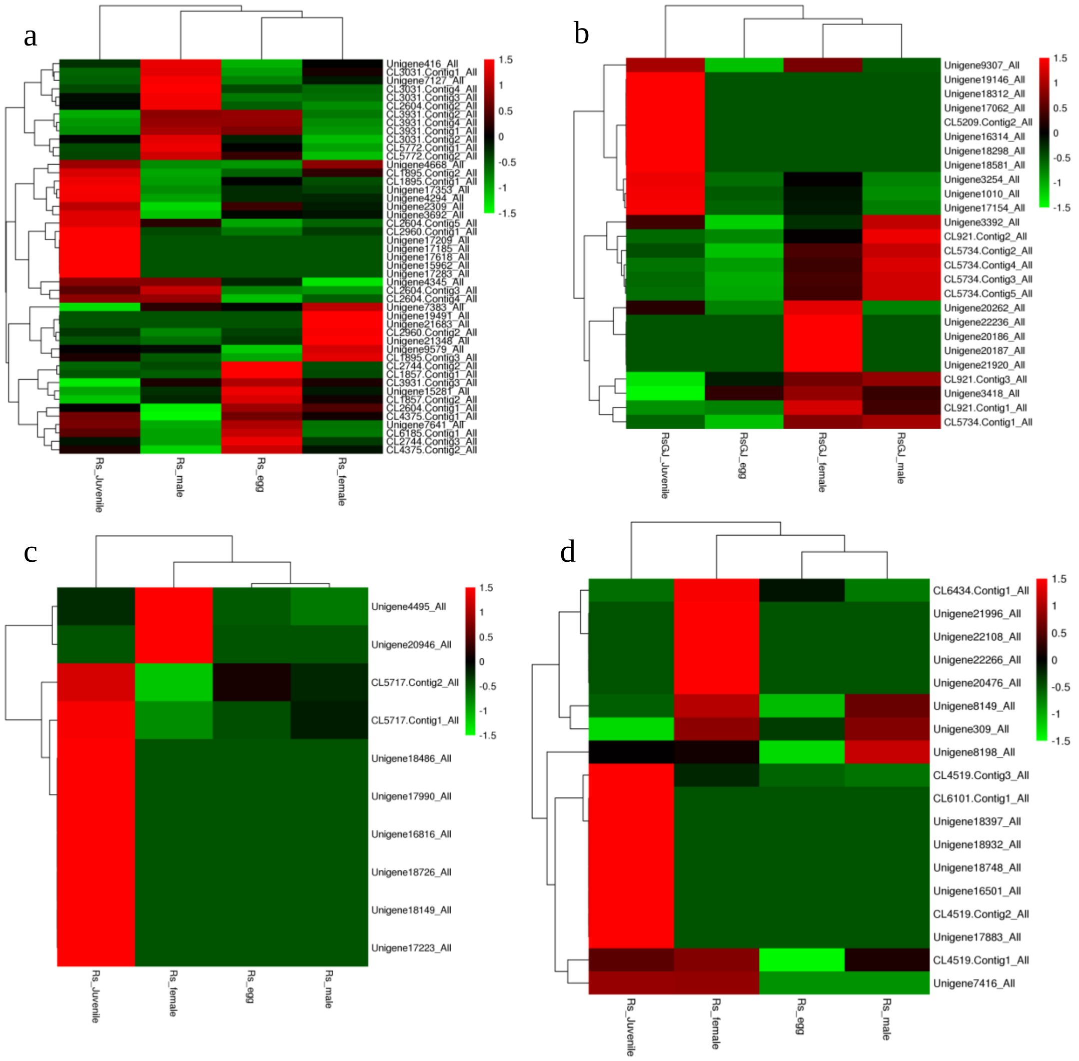

Figure S7 Heatmap of putative antioxidant genes (FDR<10-5) expression in *Radopholus similis*.

(a) Heatmap of putative GST. (b) Heatmap of putative SOD. (c) Heatmap of putative peroxiredoxin. (d) Heatmap of putative glutathione peroxidase. Expression level for each gene was presented by normalized FPKM value. Red signifies an increase in expression and green, a decrease in expression. Heatmap scale bars indicate log2-fold changes.
